# A Case Report of Monkeypox in an Adult Patient from Italy: Clinical and Dermoscopic Manifestations, Diagnosis and Management

**DOI:** 10.3390/vaccines10111903

**Published:** 2022-11-10

**Authors:** Ilaria Proietti, Paolo Emilio Santoro, Nevena Skroza, Tiziana Tieghi, Nicoletta Bernardini, Ersilia Tolino, Agnieszka Ewa Dybala, Antonio Di Guardo, Alessandra Rallo, Marco Di Fraia, Maria Francesca Rossi, Martina Vitiello, Umberto Moscato, Giovanni Pellacani, Miriam Lichtner, Concetta Potenza

**Affiliations:** 1 Department of Medical-Surgical Sciences and Biotechnologies, Dermatology Unit “Daniele Innocenzi”, Sapienza University of Rome, Polo Pontino, Fiorini Hospital, 04019 Terracina, Italy; 2 Department of Health Science and Public Health, Catholic University of the Sacred Heart, 00168 Roma, Italy; 3 Department of Neurosciences Public Health and Organs of Sense, Infectious Diseases Unit, Sapienza University of Rome, Santa Maria Goretti Hospital, 04100 Latina, Italy; 4 Dermatology Clinic, Department of Clinical Internal, Anesthesiological and Cardiovascular Sciences, Sapienza University of Rome, 00161 Rome, Italy

**Keywords:** Monkeypox, vaccine, dermoscopy, epidemy, Orthopoxvirus, dermatologic symptoms

## Abstract

Monkeypox infection is an emerging problem and a new challenge for modern medicine. With an increasing number of new cases worldwide, new data regarding the clinical manifestations, characteristics of the patients, risk factors and treatment options are coming to light. Knowing more about the disease will allow to elaborate new helpful methods to facilitate its diagnosis. Special attention should be paid to the careful dermatologic and dermoscopic examination of the patient. The analysis of available data also suggests possible strategies for the prevention of Monkeypox virus spread; the vaccine against Smallpox seems to be an effective solution. This case report describes the diagnostic approach and management of a non-vaccinated adult patient with several risk factors and a history of sexually transmitted disease. The patient had no history of travel abroad. Even though a clinical diagnose of Monkeypox can be challenging due to its similarities with skin rashes caused by other Orthopoxviral infections, there are fine differences between the rashes which can be helpful in their differentiation, although laboratory analysis is required for a definitive identification. A careful study of the characteristics of the rash, such as diameter, its presence on palms and soles and its evolution in time, provided important clues for the diagnosis of Monkeypox infection. The lack of vaccinations in the history of the patient was another crucial finding in the diagnostic process.

## 1. Introduction

Monkeypox virus is a zoonotic virus which belongs to the Orthopoxvirus genus of the *Poxviridae* family, similar to Variola and Vaccinia [1]. Monkeypox virus was first isolated in 1958 in *Macaca fascicularis* lab monkeys. Two types of the pathogen have been described: clade II (formerly West African clade) and clade I (Central African, Congo basin)-type which causes more severe disease in humans.

In the 1970s, an outbreak occurred and the Monkeypox virus (Central African type) spread initially in two African countries, Central African Republic and Democratic Republic of the Congo, during this period of time. The majority of the affected people had contact with animals. During the African outbreak in 1980s, 88% of the cases resulted from human-to-human transmission.

The first outbreak beyond Africa took place in the United States in 2003, and all the cases by that time were traced to infected rodents imported from Ghana [2,3].

In 2018, Monkeypox infection was diagnosed in the United Kingdom in two unrelated patients with a history of time spent in Nigeria. In Cameroon, close to the Nigerian border, new cases emerged in the same year. Genomic sequencing of the virus in a Cameroonian patient demonstrated a close phylogenetic relationship between pathogens from both countries. Thus, the origin of this isolate was likely an import from Nigeria.

Since then, until 2022, the infection has been detected in travelers coming from Nigeria to various countries such as Israel, Singapore, and USA. In May 2022, Italy reported the first case of Monkeypox. According to the European Centre for Diseases Prevention and Control (ECDC) report, up to 18 October 2022, 20,544 Monkeypox cases have been reported in European Countries, 888 of which were reported from Italy [4].

Most cases reported in the Central African Republic came from the areas of western and eastern rainforests. Thus, the most probable hypothesis of infections in humans is the transmission of the virus from wild animals. Dynamic changes in human activities, geographical expansion, deforestation, and land-use changes lead to increasing interactions with wild mammals. Therefore, the risk of zoonotic spillover is rising. Other contributing factors include political instabilities, as well as increasing poverty resulting in movements of populations within or outside the countries. These phenomena could have facilitated the spread of the Monkeypox virus [5].

The transmission from infected animals occurs through direct contact with human skin and mucosae. Between humans, contact with lesions, body fluids, respiratory droplets and contaminated materials are a possible cause of its dissemination. The disease can be as considered ‘sexually transmissible’ as its transmission frequently takes place during sexual intercourse. Although, the possibility of Monkeypox dissemination with semen is a matter still being studied [6].

The signs and symptoms of Monkeypox are asthenia, fever, headache, muscular aches, lymphadenopathy and skin eruption beginning 1–4 days after the fever. The rash is characterized by an evolution from macules to papule, vesicles, pustules and crusts which dry up and fall off. This rash tends to be concentrated on the face and extremities, including palms and soles. It can also affect mucosae, genitalia, and conjunctivae. The fatality ratio of Monkeypox ranges from 0 to 11%; it is higher in children and patients with comorbidities. Most commonly, the disease is self-limiting. Possible complications include secondary infections, bronchopneumonia, sepsis, encephalitis, and keratitis with vision loss [7,8]. Currently there are no licensed treatments for the infection. Vaccines against Smallpox are used to also protect against Monkeypox. Nowadays, after Smallpox eradication and the cessation of vaccination campaigns, the global risk of transmission seems to be higher, especially among young people [7,9,10]. In Italy, the Smallpox vaccine was considered an obligatory vaccine among all newborns until its suspension in 1977. In 1981 the vaccination campaign in Italy stopped [11].

Monkeypox infection can be confirmed through real-time PCR testing of swab specimens. Other laboratory tests may be helpful in differential diagnosis. In the case of Monkeypox suspicion, diagnostic investigations such as laboratory exams, dermoscopic observation and biopsy of the lesions should be performed, in order to exclude other pox-like diseases [7,9].

Herein, we describe a case of a 38-year-old man affected by the Monkeypox disease, including the diagnostic process, dermoscopic characteristics of cutaneous eruption, evolution, and its management. Even though the patient had risk factors for Monkeypox infection, he did not receive the vaccine against Smallpox. In our case, the most important differential diagnosis was Chickenpox infection, caused by the Varicella Zoster virus. The clinical manifestation of cutaneous lesions was atypical in comparison with other described cases [12].

Therefore, would like to underline the importance of careful clinical examination and dermoscopic exam which could be an extra tool to aid in the diagnosis. In light of this new infectious entity emerging in the world of medicine, special attention should be paid to patients with risk factors for Monkeypox infection, especially those non-vaccinated patients.

## 2. Case Presentation Section

A 38-year-old man was admitted to the infectious disease unit because of rectal pain, fever and vesiculopustular rash. He had been in his usual state of health until five days before the admission, when he complained of fever, rectal pain and tenesmus. During the next days the patient developed rectal bleeding, abdominal discomfort and vesicular lesions on the trunk, arms, legs, palms, soles, and face. He had a history of gonorrhea five years before, and hemorrhoidectomy when he was sixteen. He worked as a choreographer, and he attended several shows and public events two weeks prior to the hospital admission. He denied travelling abroad, he neither smoked cigarettes nor used illicit drugs. He travelled around Italy and had had several male partners, with the use of barrier protection. Although the patient had several risk factors, he was not vaccinated with the Smallpox vaccine. On examination, his temperature was 36.1 °C, the blood pressure 130/70 mmHg, the pulse 70 beats per minute and the oxygen saturation 99% when he was breathing ambient air. There was proctitis with severe tenesmus that precluded either digital rectal examination or anoscopy; the perianal skin was normal.

**Figure 1:**
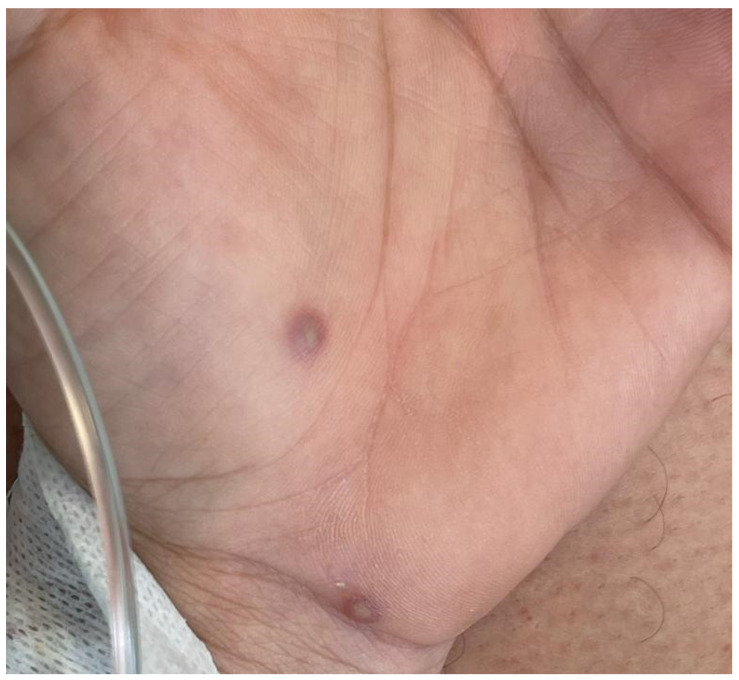
Well-circumscribed erythematous papulopustular lesions of the palm.

Skin examination showed well-circumscribed erythematous papulopustular lesions involving the trunk, arms, legs, palms, soles, and face. These had an average of 5 mm in diameter and some of them were covered by sero-hematic crusts. The lesions presented at different stages of development with bumps, blisters, and scabs existing at the same time. The mucosae were uninvolved (Figure 1).

**Figure 2:**
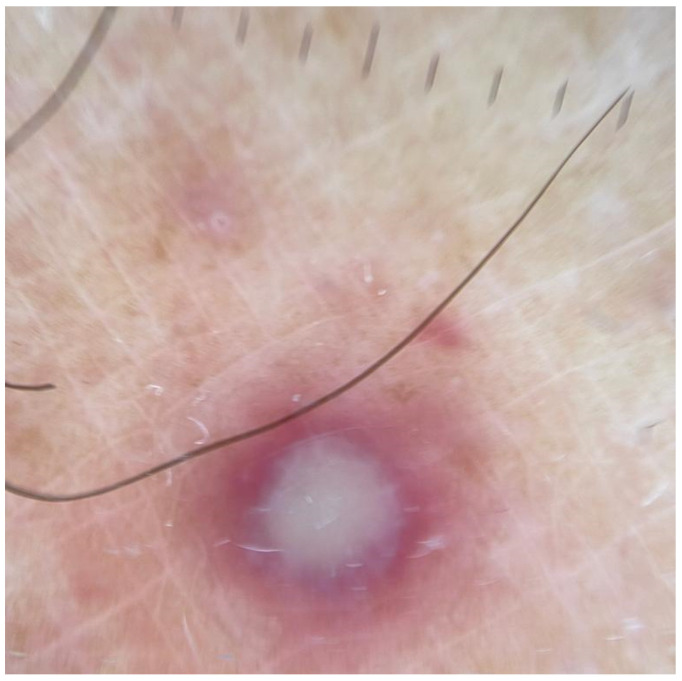
Dermoscopic examination of pustular lesion: central pale yellow opaque structureless area with ill-defined borders on the light erythematous rim in the background.

Dermoscopic examination of the pustular lesions revealed a central pale yellow opaque structureless area with ill-defined borders on the light erythematous rim in the background. Some of the lesions presented central erosion and brown-red peripherical globules (Figure 2).

No lesions in the mouth and in the external genitalia were revealed. Neither axillar nor inguinal lymphadenopathy were appreciated.

An abdominal ultrasound was normal except for intestinal bloating. The complete blood cell count was normal, as were blood levels of electrolytes, kidney function and liver function tests; C-Reactive Protein was 6.71 mg/dL (normal values < 0.5 mg/dL). He tested negative for SARS-CoV-2 infection. Blood test for syphilis, HIV, HCV, HBV were negative, as well as tests of rectal specimens for Neisseria gonorrhoeae and Chlamydia trachomatis nucleic acid. Serologic analysis for HSV-1, HSV-2, EBV, CMV, VZV and morbillivirus were consistent with previous infections. Swab specimens obtained from skin lesions and the throat were sent to the INMI “Lazzaro Spallanzani” Laboratory and real-time PCR testing detected the presence of the Monkeypox virus, West African clade. Treatment with ceftriaxone and azithromycin was started; stool softeners, morphine and beclomethasone dipropionate were administered by rectal enema. During the hospital stay, no additional skin lesions appeared, the rectal pain improved, and the fever began to abate. On the tenth hospital day, all the lesions had scabbed and fallen off, the patient was no longer considered infectious and was discharged.

## 3. Discussion

Human Monkeypox is an emerging viral zoonosis which is becoming a new challenge for modern medicine. The name Monkeypox came to light for the first time in the record from 1958, when two outbreaks of a pox-like disease took place in colonies of laboratory monkeys. Despite the name, the real source of the disease is still unknown. Still, non-human primates and African rodents might harbor the virus and infect people [13].

The transmission of the virus between humans through contact with lesions, bodily fluids, respiratory droplets and contaminated materials is well known. There is a high incidence of Monkeypox among men having sex with men, people with multiple sexual partners and those who practice condomless sex, which suggests that semen could be another vehicle for the virus. The available data demonstrate the presence of viral DNA and high quantification cycle in the seminal fluid of infected patients. Although, these findings cannot be considered definitive evidence of infectivity, they could indicate the possibility of an important viral shedding. Its efficiency in terms of transmission cannot be ruled out [6].

Even though laboratory testing is fundamental, recognition of its clinical and dermatological aspects facilitates its diagnosis and differentiation from other viruses which cause similar skin rashes, particularly Smallpox and Chickenpox. Apart from clinical examination, dermoscopic evaluation could be considered as an extra tool in this field. Since Smallpox has been eradicated, the risk of Monkeypox infection is more likely to be the cause of this type of skin rash, especially among young people, whom have not been vaccinated due to the cessation of vaccination against Monkeypox in 1981 [1,13]. In Italy, obligatory vaccination against Smallpox was suspended in 1977, thus people born after this year have never developed an immunity against the virus.

The available literature particularly underlines the differentiation between Monkeypox and Smallpox, as the greater part of the described cases come from the endemic regions for both diseases. In other countries, where the incidence of Smallpox is significantly lower, special attention should be paid to the distinction between Monkeypox and Chickenpox [13].

In this case, the first symptoms including fever, asthenia, and proctitis emerged 5 days before clinical examination. The first lesions appeared at the 4th day of illness on the face and subsequently spread in the trunk, hands, legs, and feet including palms and soles. In the case of varicella, the febrile prodrome is usually shorter (1–2 days); proctitis is not normally present. The rash does not involve the palms and soles. Lesions are usually smaller in diameter and last for less time [14].

Typically, the presence of lesions at various stages is mentioned as a distinctive characteristic of Chickenpox. However, in our case, the patient had lesions at different stages of development, including bumps, blisters, and scabs existing at the same time which made the rash less typical. Another atypical finding in our case was the lack of lymphademopathy which is usually present, especially in the neck, armpit, or groin [15].

Since dermoscopic examination is an important part of the diagnosis of dermatologic pathologies, in our case, it has revealed certain peculiarities, such as a central pale yellow opaque structureless area with ill-defined borders on the light erythematous rim in the background. The available literature also highlights the lack of vascular structures [12,16]. In our case, we found the case of palmoplantar lesions especially helpful, as in other localizations its characteristics resemble Chickenpox eruption: erythematous and yellowish background with regular margins. As a consequence, it may be helpful in distinguishing the lesions from herpes virus infections, molluscum contagiosum, orf and milker’s nodules.

Another tool which could be used in doubtful cases or in case of the unavailability of the PCR test process is histologic and immunohistochemical investigation. In case of vescicopapular lesions, these may show acanthosis, individual keratinocyte necrosis, and basal vacuolization, with a superficial and deep perivascular lymphohistiocytic infiltration in the dermis. Lesions at the vesicular stage present with spongiosis along with reticular and ballooning degeneration. Full-thickness epidermal necrosis and keratinocyte degeneration can be found with adjacent acanthosis. There may be an infiltration of lymphocytes, neutrophils, and rare eosinophils. The abovementioned features could possibly correspond to the whitish halo seen in dermoscopic examination. The keratinocytes may display an eosinophilic ‘ground glass’ appearance of the nucleus; some of them containing eosinophilic cytoplasmic inclusions (so-called Guarnieri bodies). In the dermis, full-thickness infiltration composed of lymphocytes, neutrophils, and eosinophils in a perieccrine and perivascular distribution may be noticed. Dermal vessels display some features of vasculitis with an angiotropic lymphocytic infiltration. On immunohistochemistry, the lymphocytic infiltration seems to consist predominantly of T cells with CD4^+^ and CD8^+^ elements. The CD8^+^ cells are mainly seen in the angiotropic context [12,17].

At the moment there are no specific treatments for Monkeypox. Although, given its similarities to the Smallpox virus, antiviral drugs developed to protect against Smallpox may be used to treat Monkeypox effectively [13].

Tecovirimat is approved by the Food and Drug Agency for the treatment of human Smallpox in adults and children. Nevertheless, its use for other Orthopoxvirus infections, including Monkeypox, is not approved by the FDA. The Centers for Disease Control and Prevention (CDC) holds a protocol (“compassionate use”) that allows the use of tecovirimat in the treatment of non-variola Orthopoxvirus infections, in adults and children of all ages [13,18]. In 2022 tecovirimat was also licensed by the European Medicines Agency for Monkeypox basing on data in animal and human studies. It can be used to treat Smallpox, Monkeypox and Cowpox, the infections caused by viruses belonging to the *Orthopoxvirus* family. It is also used to treat complications following vaccination against Smallpox. Still, it is not widely available [10]. Brincidofovir is another drug approved for the treatment of Smallpox which in animal models demonstrated efficacy against Monkeypox which could be a possible therapeutical option in the future [19].

In July 2022, European Medicines Agency (EMA) has released a recommendation to extend the indication of the Smallpox vaccine Imvanex as a prevention against Monkeypox disease. The medicine won approval in the European Union in 2013. It consists of an attenuated form of the vaccinia virus called ‘modified vaccinia virus Ankara’. The product is also considered a potential vaccine for Monkeypox because of the similarity between the Monkeypox virus and the Smallpox virus. The recommendation is based on data from diverse animal studies which manifested protection against the Monkeypox virus in non-human models vaccinated with Imvanex. These data were found by EMA to be strongly suggestive of the possible effectiveness of Imvanex in the prevention of the Monkeypox disease in humans [10].

Monkeypox is an emerging problem, therefore, physicians should remain up to date on this rapidly evolving front to quickly diagnose and start appropriate treatment. Since vaccine-induced immunity Monkeypox is decreasing in the general population, especially in most individuals under 50 years of age whom have not been vaccinated against it due to the cessation of vaccination campaigns after 1981, the risk of virus spread persists. Thus, an effective antiviral drug for clinically symptomatic patients or those who have been exposed or potentially exposed could be a solution.

The available literature proves that the Smallpox vaccine provides cross-protection against Monkeypox, as is well documented in animal models [20,21], and it is suggested by past data from Africa that it is able to prevent the disease in at least 85% of cases in humans [22]; the overall attack rate is lower against vaccinated individuals. The estimated percentage of unvaccinated patients among the Monkeypox cases reaches 80–96%. The infection can be described as ‘sexually transmissible’ and in the current outbreak it is spreading mainly through close personal contact. It may include contact with cutaneous lesions or respiratory secretions which occur during sexual intercourse [12].

Thus, the CDC advise vaccination for people with a higher risk of infection, especially these who had sexual contact with people diagnosed with Monkeypox in the past 2 weeks and multiple instances of intercourse in an area with known Monkeypox cases. Widespread vaccination during this outbreak is not recommended [1,12].

Factors which could limit further dissemination of the disease include better characterization of viral genomes in Central Africa Republic isolates, identifying animal hosts which today remain unknown, and evaluation of ethological and ecological risk factors in human infections [5].

An increased awareness of the Monkeypox infection and the modalities of its transmission among the population could be helpful in the control of its spread.

For the present time, vaccination of people at high risk of transmission of the virus seems to be an effective modality of Monkeypox prevention.

Better understanding of the disease is bringing to light new methods for its diagnosis and new treatment options which, in the near future, could change the way we perceive the Monkeypox virus.

## Data Availability

Not applicable.
